# Reactive Oxygen and Nitrogen Species on Monocyte and Macrophage Biology

**DOI:** 10.3390/antiox15030389

**Published:** 2026-03-19

**Authors:** Francisco Rafael Jimenez-Trinidad, Sofia Morini, Armanda Buffon, Andrea de Prisco, Greta Galati, Astrid de Ciutiis, Alessia d’Aiello, Francesc Jiménez-Altayó, Ana Paula Dantas, Giovanna Liuzzo, Anna Severino

**Affiliations:** 1 Department of Cardiovascular Sciences, Fondazione Policlinico Universitario Agostino Gemelli—IRCCS, 00136 Rome, Italy; 2 Department of Pharmacology, Therapeutics, and Toxicology, Universitat Autonoma de Barcelona (UAB), Cerdanyola del Valles, 08193 Barcelona, Spain; francesc.jimenez@uab.cat; 3 Department of Cardiovascular and Pneumological Sciences, Università Cattolica del Sacro Cuore (UCSC), 00136 Rome, Italy; 4 Centro de investigación Biomédica en Red de Enfermedades Cardiovasculares (CIBERCV), Instituto de Salud Carlos III, 28029 Madrid, Spain; 5 Institute of Neurosciences, Universitat Autonoma de Barcelona (UAB), 08193 Cerdanyola del Valles, Spain; 6 Division of Respiratory, Cardiovascular and Renal Pathobiology and Bioengineering, Institut d’Investigacions Biomèdiques August Pi i Sunyer (IDIBAPS), 08036 Barcelona, Spain; 7 Institut Clinic Cardiovascular (ICCV), Department of Cardiology, Hospital Clinic de Barcelona, 08036 Barcelona, Spain; 8 Laboratory of Environmental and Epigenetic Regulation of Endothelial Dysfunction, Department of Biomedical Sciences, School of Medicine and Health Sciences, Universitat de Barcelona (UB), 08036 Barcelona, Spain

**Keywords:** oxidative stress, reactive oxygen species, reactive nitrogen species, monocytes, macrophages, atherosclerosis

## Abstract

Reactive oxygen species (ROS) and reactive nitrogen species (RNS) are central regulators of monocyte and macrophage biology, shaping their survival, differentiation, migration, and effector functions. In monocytes and macrophages, ROS and RNS arise from endogenous sources, such as mitochondria, NADPH oxidases, and myeloperoxidase, and from exogenous stimuli including pathogens, damaged tissues, and environmental oxidants. These reactive intermediates converge on redox-sensitive pathways such as NF-κB, Nrf2/HO-1, mitochondrial ROS signalling, and the NLRP3 inflammasome, thereby integrating metabolic stress with inflammatory activation. Redox balance is a key determinant of macrophage polarization: heightened ROS and RNS production drives pro-inflammatory M1 programs, whereas tightly regulated oxidative signalling supports M2 phenotypes associated with tissue repair and resolution. In chronic inflammatory disorders, notably atherosclerosis, oxidative stress amplifies monocyte recruitment, foam-cell formation, plaque instability, and maladaptive immunometabolic responses. The aim of this review is to recapitulate the major sources and functions of ROS and RNS in monocytes and macrophages and to synthesize current evidence on how these pathways collectively maintain or disrupt immune homeostasis. We further highlight emerging therapeutic strategies, such as NOX inhibitors, mitochondrial-targeted antioxidants, and Nrf2 activators, that seek to restore redox balance and offer promising avenues for the treatment of cardiovascular and immune-mediated diseases.

## 1. Introduction

Monocytes and macrophages are essential components of the innate immune system, playing critical roles in host defence, tissue homeostasis, inflammation, and repair [1,2]. These cells originate from hematopoietic stem cells in the bone marrow, with monocytes circulating in the bloodstream as precursors that can be rapidly recruited to sites of infection or injury, where they differentiate into macrophages or dendritic cells [3,4]. Macrophages, in turn, exhibit remarkable functional and phenotypic plasticity, adopting distinct activation states, from classically activated (M1) macrophages with potent microbicidal activity to alternatively activated (M2) macrophages involved in tissue repair and immunoregulation, depending on microenvironmental cues [5,6]. This plasticity allows them to adapt their responses to diverse physiological and pathological contexts, making them central players in maintaining tissue integrity and coordinating immune responses [7,8].

Reactive oxygen species (ROS) and reactive nitrogen species (RNS) are chemically reactive molecules that function as key mediators at the interface between metabolism, immunity, and cellular signalling [9,10]. The principal ROS include superoxide anion (O_2_•^−^), hydrogen peroxide (H_2_O_2_), and hydroxyl radical (•OH), while the most prominent RNS is nitric oxide (NO•), which can react with superoxide to form peroxynitrite (ONOO•^−^), a highly reactive molecule with potent oxidative and nitrating capacity [11,12]. These species are continuously generated through multiple cellular sources, including mitochondrial oxidative phosphorylation and specialized enzymatic systems such as NADPH oxidases, xanthine oxidase, and myeloperoxidase [8]. Importantly, ROS and RNS are not merely toxic by-products; at physiological concentrations, they function as critical redox signalling intermediates that regulate gene expression, proliferation, differentiation, apoptosis, and immune activation [9,10].

Monocytes and macrophages are both major producers and targets of ROS and RNS, creating a dynamic interplay that shapes their function and fate [1,8]. In monocytes, ROS and RNS regulate survival, migration, and differentiation into macrophages or dendritic cells [3,13]. In macrophages, these reactive species are indispensable for effector functions, including pathogen clearance via the oxidative burst, regulation of inflammasome activation, and modulation of cytokine networks [5,8]. Moreover, the redox milieu critically influences macrophage polarization: M1 macrophages rely on robust ROS production for their pro-inflammatory activities, while M2 macrophages utilize more tightly regulated ROS signalling to support tissue repair [6,7]. When the balance between ROS/RNS production and antioxidant defences is disrupted, these molecules transition from physiological mediators to drivers of cellular dysfunction [9,13]. This phenomenon leads to oxidative damage to proteins, lipids, and nucleic acids, mitochondrial dysfunction, and disruption of redox-sensitive signalling pathways [11,12]. Consequently, monocyte and macrophage biology is significantly altered, disturbing the equilibrium between protective inflammation and maladaptive tissue injury [7,13]. Dysregulated ROS and RNS signalling underlie the pathogenesis of numerous diseases, including atherosclerosis, metabolic syndrome, neurodegeneration, chronic infections, autoimmunity, and cancer [6,8].

The aim of this review is to provide a comprehensive analysis of the dual roles of ROS and RNS in monocyte and macrophage biology. We will examine the sources and regulation of reactive species in these cells, their impact on key cellular processes, including survival, migration, differentiation, and polarization, and the consequences of redox imbalance in health and disease. Furthermore, we will discuss emerging therapeutic strategies targeting redox-immunometabolic pathways to restore immune homeostasis. By synthesizing current knowledge and highlighting unresolved questions, this review seeks to advance our understanding of redox control in innate immunity and identify novel approaches for treating inflammatory and degenerative disorders.

## 2. Sources of ROS/RNS in Monocytes and Macrophages

### 2.1. Endogenous Sources of ROS/RNS

**Figure 1:**
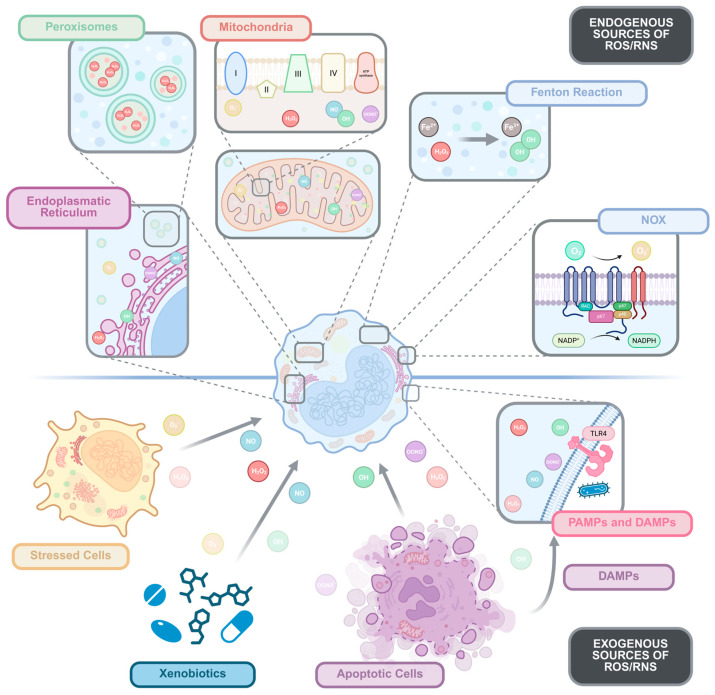
Endogenous and exogenous sources of ROS in monocytes and macrophages. The figure illustrates the various endogenous (upper panel) and exogenous (bottom panel) sources of ROS in monocytes and macrophages. Endogenous ROS production is depicted through cellular organelles, including peroxisomes, mitochondria, the endoplasmic reticulum, and the Nox complex, which generate ROS such as H_2_O_2_, O_2_•^−^, •OH, and NO•. Additionally, the Fenton reaction is shown as a mechanism for hydroxyl radical formation from Fe^2+^. Exogenous ROS sources are represented through the interaction of monocytes/macrophages with xenobiotics, apoptotic cells, and PAMPs or DAMPs, which can induce oxidative stress in the cells. Figure generated using BioRender.

Mitochondria represent a major site of ROS generation, primarily through electron leakage from complexes I and III of the electron transport chain, leading to the formation of O_2_•^−^ and its subsequent conversion to H_2_O_2_ [14,15]. Under physiological conditions, approximately 1–2% of the oxygen consumed by mitochondria is converted into ROS, mainly superoxide, which is rapidly dismutated to H_2_O_2_ by mitochondrial superoxide dismutase (MtSOD). However, under conditions of metabolic stress or mitochondrial dysfunction, such as hypoxia, hyperglycaemia, or inflammation, this production is markedly amplified [16,17,18]. The increased mitochondrial ROS not only cause oxidative damage to lipids, proteins, and DNA but also function as critical signalling molecules that modulate pathways such as NF-κB, HIF-1α, and NLRP3 inflammasome activation [19] (Figure 1). Thus, mitochondria act as central hubs where metabolic cues are translated into redox signals that influence cell fate, inflammation, and immune responses.

In addition to mitochondria, NADPH oxidase (Nox) enzymes, particularly Nox2 in phagocytes, constitute a dedicated system for rapid ROS production during the respiratory burst, a critical mechanism for microbial killing [15,20,21,22]. Nox2 is a multi-subunit enzyme complex that, upon activation, assembles in the plasma or phagosome membrane and catalyses the reduction of molecular oxygen to superoxide using NADPH as an electron donor. This process is tightly regulated by small GTPases (e.g., Rac2) and phosphorylation events, allowing for rapid and localized ROS production. Unlike mitochondrial ROS, which are primarily byproducts of metabolism, Nox-derived ROS are deliberately generated for host defence and redox signalling [23,24] (Figure 1). Other Nox isoforms, such as Nox4, are also expressed in monocytes and macrophages and contribute to sustained ROS production in chronic inflammatory conditions [21,22].

Myeloperoxidase (MPO), abundantly expressed in monocytes and especially macrophages of myeloid lineage, further amplifies oxidative potential by catalysing the formation of hypochlorous acid (HOCl) and other reactive intermediates from H_2_O_2_ [25,26]. MPO is released into phagolysosomes during phagocytosis or extracellularly upon neutrophil extracellular trap (NET) formation, where it uses H_2_O_2_ and chloride ions to generate HOCl, a potent microbicidal agent [27], excessive or dysregulated MPO activity can lead to host tissue damage and contribute to the pathogenesis of chronic inflammatory diseases, including atherosclerosis and neurodegenerative disorders [28].

Furthermore, other subcellular compartments contribute to endogenous ROS generation in monocytes and macrophages. The endoplasmic reticulum (ER) is a significant source of ROS, particularly under conditions of ER stress, where the accumulation of unfolded proteins leads to hyperactivation of ER oxidoreductases such as ERO1α and protein disulfide isomerase (PDI), which generate H_2_O_2_ as a byproduct during disulfide bond formation [29,30,31] (Figure 1). ER-derived ROS can amplify inflammatory responses through activation of the unfolded protein response (UPR) and crosstalk with NOX and mitochondrial ROS pathways [32,33]. Similarly, peroxisomes contribute to cellular ROS pools through β-oxidation of fatty acids and the activity of oxidases such as xanthine oxidase, which produce H_2_O_2_. Although peroxisomes contain catalase to degrade H_2_O_2_, an imbalance between production and scavenging can result in oxidative stress and modulation of redox-sensitive signalling pathways [34].

Non-enzymatic reactions such as the Fenton reaction and auto-oxidation of small molecules also contribute to ROS generation in these cells. The Fenton reaction, in which free iron (Fe^2+^) catalyses the conversion of H_2_O_2_ into highly reactive •OH radicals, is particularly relevant in conditions of iron overload or impaired iron homeostasis [35,36] (Figure 1). Additionally, auto-oxidation of catecholamines, quinones, and other redox-active molecules can generate superoxide and semiquinone radicals, further contributing to the intracellular oxidative milieu [37].

Several of the reactions that generate ROS interact with nitrogenous intermediates to also generate RNS, a chemically diverse family of reactive molecules whose immunological roles in monocytes and macrophages are as complex and consequential as those of their oxygen-centred counterparts. The primary source of RNS in these cells is NO•, produced by inducible nitric oxide synthase (iNOS, NOS2), whose expression is robustly induced by pro-inflammatory stimuli such as LPS and IFN-γ through NF-κB-dependent transcriptional programmes [38,39]. iNOS expression and activity are, however, subject to multilayered regulation: beyond canonical cytokine-driven induction, non-canonical iNOS expression patterns have been described in macrophages across different tissues and activation states, expanding the contexts in which NO• production may occur [39]. Mitochondrial function further modulates this balance, as uncoupling protein 2 (UCP2) plays an important role in regulating NO• production in LPS-stimulated macrophages by controlling the mitochondrial membrane potential that influences iNOS-dependent signalling [40]. The convergence of ROS and RNS pathways reaches its most consequential expression in the formation of ONOO•^−^, generated by the near-diffusion-limited reaction between O2•^−^ and NO•. Peroxynitrite is a potent oxidizing and nitrating species that represents one of the most cytotoxic molecules produced by macrophages: it is capable of oxidizing thiols, nitrating tyrosine residues, damaging DNA, and inactivating key metabolic enzymes in invading pathogens, making it a frontline cytotoxic effector in antimicrobial defence [41]. Notably, iNOS itself can directly generate both superoxide and peroxynitrite under conditions of substrate or cofactor limitation, further amplifying the oxidative-nitrosative burst at sites of inflammation [42]. The production of superoxide, NO•, and peroxynitrite by macrophages is dynamically regulated by oxygen tension, with physiological hypoxia significantly modulating the balance between these species and therefore the nature of the redox signal delivered to pathogens and surrounding tissue [43].

Importantly, significant differences exist between circulating monocytes and tissue-resident macrophages in the magnitude, regulation, and functional consequences of ROS generation. Circulating monocytes rely predominantly on mitochondrial metabolism and inducible Nox2 activity, providing a rapid but transient oxidative response upon activation [15]. Monocytes exhibit higher basal mitochondrial respiration and generate ROS mainly through oxidative phosphorylation, which allows them to respond quickly to pathogens but limits the duration of ROS production due to energy constraints and feedback regulatory mechanisms. In contrast, tissue-resident macrophages, particularly those embedded within inflamed or diseased tissues, exhibit sustained and context-dependent ROS production [44]. This is driven not only by their higher content of MPO and more robust Nox2 activity but also by profound metabolic reprogramming. For instance, M1 macrophages shift toward enhanced glycolysis (the Warburg effect), which supports rapid ATP generation and Nox2-mediated ROS production, whereas M2 macrophages rely more on mitochondrial oxidative metabolism to sustain long-term anti-inflammatory and tissue-repair functions [45,46].

### 2.2. Exogenous Triggers of ROS/RNS

Monocytes and macrophages are also exposed to ROS and RNS derived from the extracellular milieu, which significantly contribute to the redox environment in which these cells operate. Environmental toxins, pollutants, and xenobiotics can induce oxidative stress through both direct radical generation and indirect effects on cellular metabolism. For instance, airborne particulate matter (PM2.5), ozone, and heavy metals such as cadmium and mercury can directly generate free radicals or disrupt mitochondrial function, leading to increased ROS production [47,48]. Additionally, certain xenobiotics are metabolized by cytochrome P450 enzymes into reactive intermediates that redox cycle, continuously producing superoxide and hydrogen peroxide [49]. These environmental insults not only cause direct cellular damage but also prime innate immune cells for exaggerated responses to subsequent stimuli, thereby amplifying inflammation and oxidative stress in a feed-forward loop [50].

Pathogen-associated molecular patterns (PAMPs), such as lipopolysaccharide (LPS), bacterial lipopeptides, and viral nucleic acids, not only trigger innate immune responses but also stimulate Nox-dependent ROS production and inducible nitric oxide synthase (iNOS) activity, amplifying RNS generation. Upon recognition by pattern recognition receptors (PRRs) like Toll-like receptors (TLRs), PAMPs activate downstream signalling cascades (e.g., NF-κB and MAPK pathways) that upregulate the expression and assembly of Nox2 complexes, leading to a robust respiratory burst [51,52]. Concurrently, iNOS is induced in macrophages, producing large amounts of NO•, which can react with superoxide to form ONOO•^−^, a potent RNS capable of nitrating tyrosine residues and damaging cellular components [53] (Figure 1). This coordinated production of ROS and RNS is essential for pathogen clearance but, when dysregulated, contributes to tissue injury and chronic inflammation [54].

Furthermore, tissue injury and necrosis release damage-associated molecular patterns (DAMPs) and pro-oxidant molecules, including free heme and oxidized lipids, which further exacerbate ROS/RNS production in recruited immune cells. DAMPs such as high-mobility group box 1 (HMGB1), heat shock proteins, and extracellular ATP act as endogenous danger signals that activate PRRs on monocytes and macrophages, leading to Nox activation and mitochondrial ROS generation [55,56]. Free heme, released from haemoglobin during haemolysis or tissue damage, is a potent pro-oxidant that catalyses Fenton-like reactions, generating •OH radicals and inducing lipid peroxidation [57] (Figure 1). Similarly, other signals of damage and dysregulation, such as oxidized low-density lipoproteins (oxLDL) and other oxidized phospholipids accumulate in atherosclerotic lesions and other inflammatory sites, where they bind to scavenger receptors on macrophages, triggering intracellular ROS production and foam cell formation [58].

In addition to these sources, ROS and RNS can be generated by neighbouring cells in a paracrine manner, further modulating the redox microenvironment. For example, activated neutrophils and eosinophils release extracellular ROS and MPO-derived oxidants during inflammation, which can diffuse to nearby monocytes and macrophages, influencing their activation state and function [59]. Similarly, endothelial cells and fibroblasts produce ROS in response to cytokines and mechanical stress, contributing to the oxidative milieu in tissues such as the vascular wall or the myocardium [60,61] (Figure 1).

## 3. Oxidative Stress and Monocyte Biology

**Figure 2:**
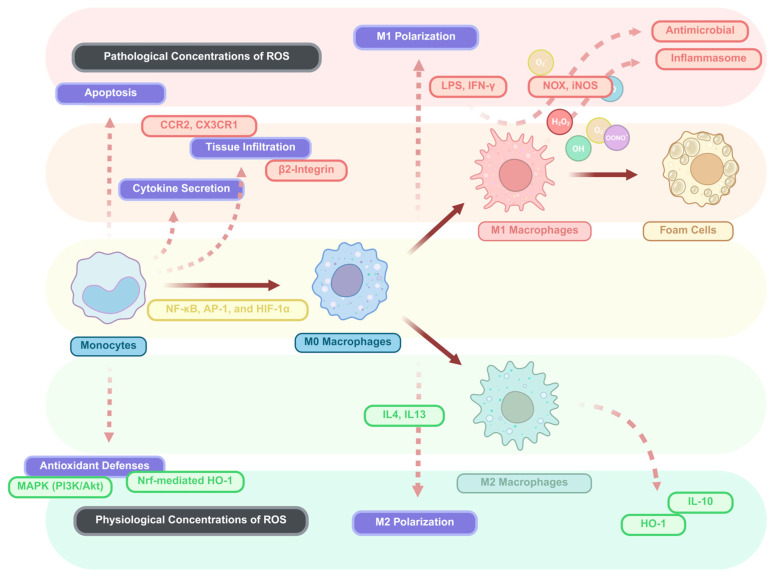
Roles of ROS and RNS in monocyte and macrophage biology at physiological and pathological concentrations. The figure depicts the roles of ROS in monocyte—to—macrophage differentiation and inflammation under both physiological and pathological conditions. At pathological concentrations of ROS/RNS (top panel), these species influence processes such as apoptosis, M1 polarization, and the activation of antimicrobial responses. Key mediators like LPS, IFN—γ, and Nox are involved in M1 macrophage polarization, which leads to the release of pro—inflammatory cytokines and the formation of foam cells. In contrast, at physiological ROS/RNS concentrations (bottom panel), ROS/RNS are involved in M2 polarization and the promotion of anti-inflammatory responses, with the activation of antioxidant defences such as MAPK (PI3K/Akt) and Nrf-mediated HO—1 expression. The shift between these ROS/RNS levels influences monocyte differentiation, cytokine secretion, and macrophage polarization, with distinct outcomes in inflammation and tissue response. Figure generated using BioRender.

Monocytes are highly dynamic cells whose survival, migratory capacity, and differentiation are profoundly influenced by the balance of ROS and RNS. As short-lived circulating precursors, they are particularly sensitive to fluctuations in redox status, which act as key determinants of their fate and functional programming [62,63]. This sensitivity stems from their relatively limited antioxidant defences compared to other immune cells, rendering them vulnerable to oxidative stress while also allowing redox signals to potently modulate their responses [63,64],

ROS and RNS play a dual role in controlling monocyte viability. At physiological concentrations, they function as signalling molecules that promote adaptive stress responses, enhancing resistance to metabolic fluctuations and inflammatory stimuli [62,65]. For instance, controlled redox signalling can activate the Nrf2 pathway, leading to the upregulation of antioxidant enzymes such as heme-oxygenase-1 (HO-1), which contributes to cytoprotection. In line with this concept, reduced HO-1 expression has been observed in pro-inflammatory monocyte subpopulations from patients with lupus nephritis, suggesting that impaired antioxidant responses are associated with heightened inflammatory activation [66]. However, excessive or sustained ROS/RNS production triggers oxidative damage to mitochondrial membranes, activation of caspase-dependent pathways, and DNA fragmentation, thereby accelerating apoptosis. This redox-dependent regulation of survival not only governs the circulating monocyte pool but also influences the availability of precursors for macrophage and dendritic cell lineages in tissues [63,67]. However, monocytes from patients with chronic granulomatous disease, which are deficient in ROS production, exhibit aberrant gene expression and increased susceptibility to apoptosis, highlighting the importance of basal ROS levels for normal monocyte homeostasis [64] (Figure 2).

Monocyte trafficking to sites of inflammation is critically modulated by ROS and RNS. Reactive species, highly expressed after cellular and tissular injury, regulate the expression and activation of adhesion molecules (e.g., integrins, selectins) and chemokine receptors (e.g., CCR2, CX3CR1), thereby shaping monocyte extravasation and tissue homing. For example, ROS-mediated oxidation of chemokines can enhance their chemotactic activity, creating gradients that facilitate monocyte recruitment [68,69]. Additionally, ROS generated during monocyte adhesion to endothelial cells or extracellular matrix components serve as secondary messengers that activate integrins and promote firm adhesion [62]. NO• and its derivatives further influence vascular tone and endothelial activation, indirectly regulating monocyte adhesion and transmigration [70]. Overexpression of uncoupling protein 2 (UCP2) in monocytes reduces mitochondrial ROS production and inhibits β2 integrin-mediated adhesion and trans-endothelial migration, suggesting a direct link between mitochondrial metabolism and migratory capacity [69]. Moreover, in inflammatory conditions such as sepsis, modulation of oxidative metabolism in monocytes correlates with altered expression of surface receptors and impaired migratory function, underscoring the clinical relevance of redox regulation in monocyte trafficking [71] (Figure 2).

Monocyte function is not only shaped by redox signalling per se but is also intimately linked to the metabolic state of the cell, which in turn feeds back onto ROS and RNS production. Monocytes are metabolically flexible cells capable of dynamically rewiring their bioenergetics in response to inflammatory stimuli, and this metabolic reprogramming is tightly coupled to their functional outputs [72,73]. Glycolysis emerges as a particularly critical metabolic pathway in this context: it is required for LPS-induced activation and adhesion of human CD14^+^CD16^−^ monocytes, and its upregulation supports the rapid energetic demands of inflammatory responses [74]. Beyond acute activation, glucose metabolism also controls monocyte homeostasis and migratory capacity under steady-state conditions, although its contribution to pathological processes such as atherosclerosis appears to be more nuanced and context-dependent [75]. Importantly, elevated glycolytic activity is not without consequences for monocyte fate decisions: in tuberculosis, excessive glycolytic metabolism in monocytes limits the generation of HIF-1α-driven migratory dendritic cells, illustrating how metabolic programming can constrain differentiation trajectories with direct implications for antimicrobial immunity [76,77]. At a broader level, ROS function as integral components of metabolic and inflammatory signalling networks, serving as both products and regulators of metabolic flux [78,79]. This bidirectional relationship is further modulated by mTOR, which acts as a metabolic checkpoint that restrains LPS-induced monocyte inflammatory and procoagulant responses by limiting metabolic reprogramming [80]. Notably, ageing introduces an additional layer of complexity, as classical monocytes from older adults maintain glycolytic metabolism ex vivo but exhibit selectively impaired late inflammatory responses, suggesting that age-related changes in metabolic-redox coupling may contribute to immunosenescence [81].

The differentiation of monocytes into macrophages or dendritic cells is tightly controlled by local microenvironmental signals, many of which converge on redox-sensitive pathways. ROS influence lineage specification by modulating transcription factors such as NF-κB, AP-1, and HIF-1α, as well as epigenetic regulators of gene expression [82,83]. In oxidative microenvironments, monocytes are more likely to differentiate toward pro-inflammatory (M1-like) macrophages, characterized by increased production of cytokines and ROS [84]. Conversely, balanced redox conditions favour alternative (M2-like) or tolerogenic differentiation pathways, associated with tissue repair and resolution of inflammation [82,85]. RNS, particularly NO•, can further regulate this process by nitrosylation-related signalling proteins and influencing cytokine-driven differentiation programs. For instance, NO• has been shown to inhibit ROS production in Mycobacterium tuberculosis-infected monocytes, potentially altering their differentiation and antimicrobial responses [86]. Otherwise, oxidized proteins in the microenvironment can differentially affect monocyte maturation and activation, with some oxidized species promoting pro-inflammatory phenotypes while others induce tolerance [85]. (Figure 2).

Together, these mechanisms highlight ROS and RNS as key modulators of monocyte biology. By orchestrating survival, migration, and differentiation, reactive species not only determine the functional output of circulating monocytes but also set the stage for downstream macrophage and dendritic cell responses within tissues [87,88]. Dysregulation of redox balance in monocytes is implicated in various pathological conditions, including chronic inflammatory diseases, atherosclerosis, and infections. For example, in atherosclerosis, Nox4-derived ROS in monocytes promote macrophage death and contribute to plaque instability [66,84] while in malaria, xanthine oxidase-produced ROS drive inflammatory responses that exacerbate disease severity [88]. However, even in states of immune tolerance, such as endotoxin tolerance, monocytes retain the ability to phagocytose bacteria and generate ROS, suggesting that redox regulation is preserved in certain adaptive responses [89].

## 4. Oxidative Stress on Macrophage Function and Polarization

Macrophage polarization is a highly plastic and context-dependent process in which ROS and RNS serve as pivotal modulators. Depending on the redox milieu and the integration of environmental cues, macrophages acquire phenotypes ranging from classically activated, pro-inflammatory M1 cells to alternatively activated, anti-inflammatory and reparative M2 cells (Although macrophage activation is currently understood as a continuum of functional states rather than a strict dichotomy, the classical M1/M2 terminology is used here when referring to studies that originally adopted this classification). This plasticity allows macrophages to adapt their functional responses to diverse microenvironmental signals, with ROS and RNS fine-tuning macrophage activation through redox-sensitive signalling pathways [90].

Increased ROS are strongly associated with the acquisition and maintenance of the M1 phenotype. Pro-inflammatory stimuli such as LPS and interferon-γ (IFN-γ) trigger NOX and iNOS activity, leading to robust production of O2•^−^ and NO• [26,42]. These reactive intermediates amplify antimicrobial activity, enhance antigen [41,91]. Mitochondria-derived O2•^−^, produced by reverse electron transport at complex I is essential for IL-1β release during NLRP3 inflammasome activation in M1 macrophages [92]. However, ROS does not only participate in acute inflammatory responses but also contributes to deleterious chronic inflammation. In this sense, MPO-mediated oxidative stress have been shown to prolong the pro-inflammatory properties of M1 macrophages, driving a disbalanced pro-inflammatory condition [93].

In contrast, M2 polarization, typically induced by IL-4 or IL-13, is characterized by a metabolic shift toward oxidative phosphorylation and a relative decrease in oxidative burst activity. In this setting, ROS levels are tightly regulated to support tissue remodelling and repair while minimizing collateral damage. For instance, IL-10 secretion in M2 macrophages depends on O2•^−^ production by mitochondrial complex III, highlighting a redox-dependent mechanism that supports anti-inflammatory functions [92]. Moreover, HO-1, an antioxidant enzyme induced during macrophage differentiation, helps maintain redox balance in M2 macrophages, protecting them from excessive oxidative stress [94]. Thus, while M1 macrophages rely on high ROS levels for host defence, M2 macrophages utilize controlled ROS signalling for resolution and repair (Figure 2).

ROS and RNS exert direct control over cytokine networks that define macrophage functional states. In M1 macrophages, elevated ROS promote the activation of NF-κB and inflammasome pathways, driving the production of IL-1β and TNF-α [95]. These cytokines reinforce the inflammatory cascade, recruit additional immune cells, and enhance microbial clearance. H_2_O_2_ in particular, stimulates macrophages and monocytes to actively release HMGB1, a pro-inflammatory DAMP that amplifies immune responses [96]. Conversely, in M2 macrophages, redox-sensitive pathways favour the expression of anti-inflammatory cytokines such as IL-10, which dampen inflammation and promote resolution [92,97]. NO• and ONOO•^−^ further shape these responses by post-translationally modifying signalling proteins, thereby fine-tuning cytokine output. For example, ONOO•^−^ can nitrate tyrosine residues in key signalling molecules, altering their function and modulating cytokine production [98] (Figure 2).

The redox-dependent regulation of macrophage polarization has profound consequences for the balance between inflammation and healing. Persistent ROS-driven M1 polarization contributes to chronic inflammation, tissue injury, and the pathogenesis of diseases such as atherosclerosis and autoimmune disorders. For instance, in atherosclerosis, ROS mediate cyclooxygenase-2 induction during monocyte-to-macrophage differentiation, promoting plaque instability [93,99]. Similarly, in lupus, NCF1-dependent ROS production regulates plasmacytoid dendritic cell development and functions, protecting against autoimmunity [100].

Conversely, controlled modulation of ROS and RNS in favour of M2-like states facilitates the clearance of apoptotic cells, deposition of extracellular matrix, angiogenesis, and tissue regeneration. H_2_O_2_, for example, stimulates macrophage vascular endothelial growth factor release, promoting angiogenesis and tissue repair. Moreover, macrophage-derived O2•^−^ production following skeletal muscle injury supports antioxidant responses and tissue regeneration [101,102]. Dysregulation of these processes, whether through excessive oxidative stress or inadequate redox signalling, can impair the resolution of inflammation and predispose to pathological remodelling [103,104].

However, ROS levels are not strictly coupled to a single polarization outcome, and the relationship between oxidative signals and macrophage phenotype is considerably more nuanced than a simple M1-promoting role. Emerging evidence demonstrates that ROS can also actively drive M2 polarization and the emergence of tumour-associated macrophages depending on the source, compartment, and intensity of the oxidative signal: mitochondrial ROS in particular have been shown to play a critical role in alternative macrophage activation, with distinct mitochondrial ROS-dependent pathways, including RBPJ knockdown-driven Notch1-Jagged1-Hes1 signalling and IL-25-induced AMPK-associated mitophagy, promoting M2 polarization in inflammatory and tumour microenvironments [90,105,106]. Conversely, ROS can also reprogram macrophages toward M1 states through non-canonical mechanisms, such as the ROS-ATM-Chk2 axis, which mediates metabolic and cell cycle reprogramming during M1 polarization independently of classical cytokine-driven pathways [107].

The delicate balance between ROS production and antioxidant defences is central to macrophage homeostasis, requiring continuous adaptation to both endogenous and exogenous oxidative challenges. Prolonged exposure to H_2_O_2_, common in associated with multiple comorbidities and aging, induces catalase upregulation in macrophages, reflecting an adaptive antioxidant response that limits oxidative stress-induced damage [103]. Consistent with this notion, long-lived p66Shc knock-out mice display reduced O_2_•^−^ production in macrophages, which correlates with decreased oxidative damage and enhanced organismal longevity [104]. Importantly, this redox balance is not only relevant for cellular homeostasis but also critically shapes macrophage effector functions during infection. In the context of Trypanosoma cruzi infection, macrophage-derived ROS and RNS are essential for microbial killing; however, the sustained oxidative pressure exerted by the host has driven the evolution of parasite antioxidant strategies, including the expression of peroxiredoxins that detoxify ONOO•^−^ and dampen host-mediated oxidative damage [98,108].

As phagocytes, macrophages rely critically on ROS and RNS to execute one of their most fundamental functions: the engulfment and destruction of pathogens, apoptotic cells, and debris. ROS production is not merely a byproduct of phagocytosis but an active and regulated component of the phagocytic process itself, with NADPH oxidase-derived superoxide serving as a central effector of microbial killing within the phagosome [109,110]. Indeed, phagocytosis itself induces O2•^−^ formation, which can in turn trigger apoptotic programmes in macrophages, coupling phagocytic activity to cell fate decisions [111]. The relationship between ROS and phagocytosis is, however, bidirectional and context-dependent: while ROS are required for the phagocytosis of myelin by macrophages in neuroinflammatory settings, macrophage-derived oxygen radicals can also exert divergent, and sometimes inhibitory, effects on phagocytic capacity depending on the nature and magnitude of the oxidative burst [112,113]. Mitochondria further contribute to this regulation, as the interaction between mitochondrial ROS, HO-1, and nitric oxide synthase stimulates phagocytic activity, while mitochondrial dysfunction, as observed in COPD macrophages, is associated with defective bacterial phagocytosis, underscoring the metabolic-redox basis of phagocytic competence [114,115]. Macrophage polarization state adds yet another layer of complexity, as M1 and M2 macrophages display distinct phagocytic capacities and ROS profiles that are regulated independently of receptor surface expression, and different macrophage phenotypes exhibit divergent radical-generating activities that correlate with their functional specialization [116,117]. Finally, the link between autophagy and phagocytosis, processes that share molecular machinery and are both sensitive to ROS, further integrates oxidative signalling into the broader programme of macrophage-mediated clearance and homeostasis [118].

Precisely in the context of phagocytosis, peroxynitrite emerges as a central RNS effector, acting within the phagosome compartment as one of the most potent microbicidal species generated by macrophages. Intraphagosome ONOO•^−^, formed by the rapid combination of NADPH oxidase-derived O2•^−^ and iNOS-derived NO•, constitutes a primary cytotoxic mechanism against internalized pathogens: in the context of Trypanosoma cruzi infection, intraphagosome ONOO•^−^ has been directly demonstrated to mediate oxidative killing of the parasite, with the infectivity of the organism depending critically on the expression of microbial peroxiredoxins capable of detoxifying this species, illustrating the evolutionary arms race between host RNS-based defence and pathogen antioxidant adaptation [98]. The in vivo relevance of peroxynitrite-mediated microbicidal activity is further supported by the detection of protein nitration and hydroxylation products, hallmarks of ONOO•^−^ chemistry, in macrophages engaged in phagocytosis, confirming that peroxynitrite formation is not merely a biochemical possibility but an active component of the oxidative burst in living tissue [91]. Beyond direct pathogen killing, peroxynitrite generated during or after phagocytosis can also modulate downstream inflammatory signalling: the ONOO•^−^/PKR axis has been shown to activate the NLRP3 inflammasome in a manner that amplifies the inflammatory response initiated by phagocytic events, coupling RNS chemistry to inflammasome-driven IL-1β processing and secretion [119]. The dual origin of peroxynitrite from iNOS, which can simultaneously generate both NO• and O2•^−^ under conditions of cofactor limitation, means that a single enzymatic source can self-sustain the nitro-oxidative burst within the phagosome independently of NADPH oxidase, further underscoring the centrality of iNOS-derived RNS to macrophage microbicidal chemistry [42]. Finally, the immunometabolic consequences of NO• production during phagocytic activation extend well beyond the phagosome itself: NO• orchestrates a broad metabolic rewiring in M1 macrophages by targeting mitochondrial aconitase 2 and pyruvate dehydrogenase, inhibiting the TCA cycle and redirecting carbon flux in ways that sustain the pro-inflammatory state and support ROS and RNS production over the course of an immune response [120,121].

ROS and RNS do not only shape macrophage life and function, but also critically determine the mode of their death, with oxidative stress serving as a key gatekeeper across multiple regulated cell death pathways. At the broadest level, ROS act as central mediators that can channel macrophages towards apoptosis, ferroptosis, pyroptosis, necroptosis, or paraptosis depending on the intensity, source, and context of the oxidative signal [122]. Ferroptosis, an iron-dependent form of regulated cell death driven by lipid peroxidation, is particularly relevant in macrophages: in atherosclerosis, macrophage iron overload and ferroptosis contribute to plaque progression and instability, while in apical periodontitis, macrophage ferroptosis driven by the NRF2/FSP1/ROS axis facilitates bone loss, illustrating the pathological consequences of this oxidative death modality [123,124,125]. Necroptosis, a pro-inflammatory programmed necrosis, is likewise tightly coupled to ROS: mitochondrial ROS prime the hyperglycaemic shift from apoptosis to necroptosis, and ROS-mediated M1 polarization–necroptosis crosstalk amplifies inflammatory tissue damage in multiple pathological contexts [126,127]. Furthermore, mitochondrial ROS promote susceptibility to infection via gasdermin D-mediated necroptosis, while scavenging ROS production can prevent necroptotic injury, highlighting the therapeutic potential of targeting this axis [127,128]. Pyroptosis, characterized by gasdermin-mediated membrane pore formation and release of IL-1β and IL-18, is also deeply intertwined with ROS: mitochondrial ROS promote macrophage pyroptosis by inducing GSDMD oxidation, TRAF3 drives ROS production to amplify pyroptotic signalling via ULK1 ubiquitination, and ROS-induced pyroptosis contributes to cardiovascular and inflammatory diseases [129,130,131,132]. Conversely, activation of the NRF2 pathway by kynurenic acid suppresses ROS-driven pyroptosis in macrophages, and pharmacological inhibition of inflammasome assembly limits pyroptotic lung injury, underscoring that antioxidant programmes directly constrain pyroptotic cell death [133,134].

However, macrophages are not only passive players in the face of oxidative stress, but have evolved a remarkable array of adaptive mechanisms to defend against oxidative injury and ensure their own survival. At the transcriptional level, the Nrf2 pathway plays a central role in coordinating the upregulation of a broad cluster of oxidative stress-inducible genes, including HO-1, NQO1, and glutathione-related enzymes, enabling macrophages to mount a rapid and comprehensive antioxidant response upon oxidant challenge [135,136]. Consistent with this, glutathione has been shown to directly protect RAW 264.7 macrophages from oxidative stress-induced cytotoxicity through activation of the Nrf2/HO-1 axis, underscoring the central importance of this pathway in macrophage cytoprotection [137]. Beyond transcriptional responses, macrophages deploy additional molecular strategies to resist oxidative damage; the cytokine MIF provides protection against pro-oxidative stress-induced apoptosis, both in the context of ischemia/reperfusion injury and under conditions of general oxidative challenge, acting as an endogenous safeguard against excessive ROS-driven cell death [138,139]. Nevertheless, when oxidative stress overwhelms these defences, macrophages engage tightly regulated survival-versus-apoptosis decision programmes involving the interplay between pro-survival kinases, mitochondrial integrity signals, and caspase activation cascades [140,141].

## 5. Redox Signalling Pathways

**Figure 3:**
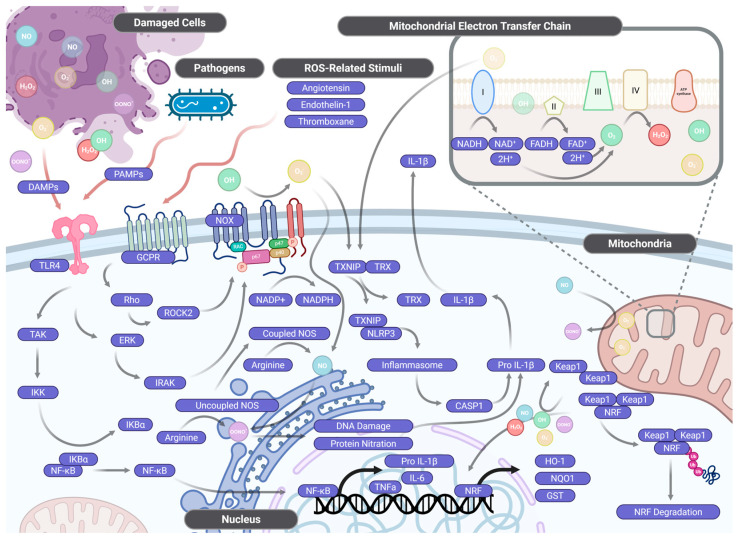
ROS/RNS-related molecular pathways. This figure illustrates the molecular pathways involved in ROS/RNS production and signalling in cells. It highlights the key components and processes initiated by ROS/RNS, such as those related to mitochondrial electron transfer, pathogen recognition, and ROS/RNS—related stimuli. The mitochondrial electron transport chain is shown as a major source of ROS/RNS, producing molecules like O_2_•^−^ and H_2_O_2_, which activate various signalling pathways. These ROS/RNS can trigger the activation of the Nox complex, GPCRs, and receptors like TLR4, leading to the phosphorylation of key proteins (e.g., RAC, p47) and the subsequent activation of NF—κB and other inflammatory signals. Additionally, under conditions of tetrahydrobiopterin deficiency or oxidative stress, eNOS uncoupling shifts nitric oxide synthase activity from NO• production toward O_2_•^−^ generation; the resulting O_2_•^−^ reacts rapidly with any available NO• to form peroxynitrite (ONOO^−^), a potent nitro—oxidative species that amplifies vascular and tissue damage. ROS/RNS also influence the TXNIP/TRX complex and inflammasome activation, which regulates the production of pro-inflammatory cytokines like IL—1β and TNF—α. These signalling cascades also involve the Keap1—NRF2 pathway, which regulates antioxidant defences such as HO—1 and NQO1. This interconnected network of pathways links ROS/RNS to inflammation, oxidative stress, and cellular response mechanisms. Figure generated using BioRender.

Redox signalling pathways in monocytes and macrophages constitute a complex network that generates and transduces oxidative and nitrosative signals into functional cellular responses. Central to this network are the NF-κB and Nrf2/HO-1 pathways, which act as counterbalancing yet interconnected regulators of inflammation and antioxidant defence. NF-κB is activated by ROS and RNS through the degradation of IκB inhibitors, leading to nuclear translocation and transcription of pro-inflammatory genes, including TNF-α, IL-6, and IL-1β. In contrast, oxidative stress induces Nrf2 to dissociate from Keap1 in the cytoplasm and translocate to the nucleus, promoting transcription of antioxidant genes such as HO-1, NADPH quinone oxidoreductase 1 (NQO1), and glutathione S-transferases (GSTs) [90,142]. The dynamic balance between NF-κB-driven inflammation and Nrf2-mediated antioxidant responses is critical for macrophage polarization and the maintenance of cellular homeostasis under oxidative stress (Figure 3).

Beyond these canonical pathways, Nox complexes represent a major source of ROS and a hub for redox signalling. The assembly and activation of Nox enzymes, particularly Nox2, are tightly regulated by phosphorylation events and protein-protein interactions. For instance, the ERK-IRAK-p67phox-Nox2 axis is activated downstream of TLR4 and TLR2 signalling, linking tissue damage and pathogen recognition to ROS production, which in turn facilitates IL-1β transcription and processing in monocytes. Similarly, while in an indirect way, the ROCK2-p22phox-p47phox module plays a crucial role in human monocytes, where ROCK2 interacts with p22phox to phosphorylate p47phox, thereby controlling Nox activation and subsequent ROS generation [143] (Figure 3). Additional regulatory mechanisms include the small GTPase Rac, which is essential for Nox2 activation, and protein kinase C (PKC), which phosphorylates p47phox to facilitate its translocation to the membrane [144,145].

Mitochondrial ROS also serve as critical signalling molecules, particularly under metabolic stress or hypoxia. Changes in mitochondrial enzymatic activities, such as those occurring during prolonged hypobaric hypoxia, can significantly alter ROS/RNS production in monocytes, influencing their survival, activation, and inflammatory responses [146]. Mitochondrial dysfunction not only amplifies oxidative stress but also impairs cellular bioenergetics, further exacerbating inflammatory processes [147,148] (Figure 3).

The NLRP3 inflammasome represents another critical redox-sensitive pathway in monocytes and macrophages. ROS, particularly those generated by mitochondria or Nox enzymes, serve as a secondary signal for NLRP3 activation, leading to caspase-1-mediated cleavage and secretion of IL-1β and IL-18 [149,150]. This pathway is tightly regulated by thioredoxin-interacting protein (TXNIP), which dissociates from thioredoxin under oxidative stress and binds to NLRP3 to promote inflammasome assembly [151] (Figure 3). Additionally, ROS can modulate autophagy, a process essential for removing damaged organelles and maintaining cellular homeostasis. Oxidative stress induces autophagy through activation of AMPK and inhibition of mTOR, while autophagy, in turn, helps mitigate oxidative damage by clearing dysfunctional mitochondria (mitophagy) and aggregated proteins [152,153].

Chemokine-mediated pathways can also induce oxidative stress in neighbouring cells; for example, MCP-1-activated monocytes generate ROS that promote apoptosis in human retinal pigment epithelium, illustrating how redox signalling can mediate cell–cell communication in pathological contexts [154]. Similarly, other cytokines such as TNF-α and IFN-γ can amplify ROS production through positive feedback loops, further enhancing inflammatory responses [155] (Figure 3).

While not direct receptors nor effectors, the MAPK family, including ERK, JNK, and p38, serves as a central hub for transducing oxidative and nitrosative signals into coordinated cellular responses. ROS can modulate MAPK activity both by direct oxidation of upstream kinases and by inhibiting phosphatases, leading to activation of downstream transcription factors such as AP-1 and ATF2. Through this central positioning, MAPKs integrate diverse stimuli, including Nox- and mitochondria-derived ROS, NLRP3 inflammasome activation, cytokines like TNF-α and IFN-γ, and chemokine-mediated intercellular signals, translating them into functional outputs such as proliferation, differentiation, apoptosis, and inflammatory gene expression. By acting as critical transducers, MAPKs not only amplify redox cues but also coordinate the crosstalk between pro-inflammatory NF-κB pathways and antioxidant Nrf2 responses, thereby shaping macrophage polarization and orchestrating context-dependent immune functions [156].

ROS do not only act directly by activating proteins but also induce post-translational modifications (PTMs) that profoundly alter protein function and ultimately determine cell fate. As highlighted by Sies, Jones, and colleagues, ROS function as pleiotropic physiological signalling agents, and their role in redox regulation extends far beyond simple oxidative damage to encompass precise, reversible modifications that fine-tune immune responses [157,158]. In monocytes and macrophages, oxidative PTMs, including sulfonylation, glutathionylation, and nitrosylation of cysteine residues, as well as carbonylation and methionine oxidation, represent a sophisticated layer of signalling that modulates the activity of key redox-sensitive proteins involved in the pathways described above [159,160]. Importantly, these oxidative modifications do not operate in isolation; rather, they are deeply intertwined with phosphorylation networks, such that changes in protein oxidation state can directly influence kinase and phosphatase activity, thereby reshaping the phosphoproteome and downstream inflammatory outputs [161]. This crosstalk between redox PTMs and phosphorylation is particularly relevant in the context of macrophage polarization, where it contributes to the dynamic regulation of NF-κB, Nrf2, and MAPK signalling [162,163,164]. While many of these modifications are reversible and serve homeostatic functions under physiological conditions [165], their dysregulation under chronic oxidative stress can drive pathological outcomes, including the aberrant activation of inflammasomes, impaired antioxidant defences, and the perpetuation of inflammatory disease states [160,166]. Together, these findings underscore that redox PTMs constitute an essential, yet underappreciated, dimension of the signalling landscape in innate immunity, integrating oxidative cues from Nox enzymes, mitochondria, and the extracellular milieu into coherent and context-dependent macrophage responses.

## 6. Effects of ROS and RNS on Monocyte and Macrophage Function in Atherosclerosis and Coronary Syndromes

Atherosclerosis and its clinical manifestations, including acute coronary syndromes, are fundamentally driven by chronic inflammation and oxidative stress, with monocytes and macrophages playing central roles in disease initiation and progression [167,168]. In the arterial wall, monocytes are recruited to sites of endothelial dysfunction, where they differentiate into macrophages and engulf oxLDL, giving rise to foam cells, the hallmark of early atherosclerotic lesions [169]. This process is critically dependent on ROS and RNS, which not only promote LDL oxidation but also modulate every aspect of monocyte and macrophage function, from recruitment and activation to apoptosis and efferocytosis [170]. Monocytes and macrophages in atherosclerotic lesions exhibit increased ROS production, which serves both as a driver of inflammation and a marker of disease activity. Clinical studies have demonstrated that ROS production by monocytes negatively correlates with disease activity in rheumatoid arthritis, a condition with increased cardiovascular risk, but positively correlates with aging and atherosclerosis progression [171,172].

**Figure 4:**
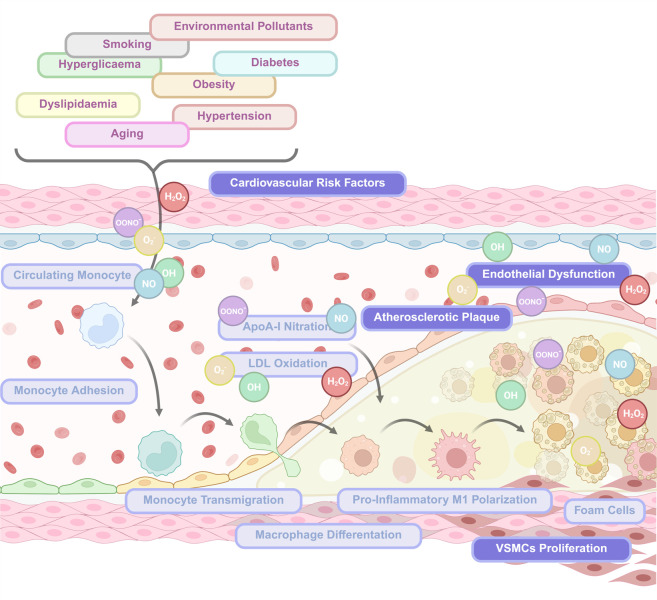
ROS/RNS modulating monocyte and macrophage behaviour in the vasculature and the atherosclerotic plaque. Cardiovascular risk factors, including aging, smoking, diabetes, obesity, hypertension, dyslipidaemia, hyperglycaemia, and environmental pollutants, promote excessive production of ROS/RNS such as O_2_•^−^, H_2_O_2_, •OH, and ONOO•^−^ within the vascular wall. Increased oxidative stress disrupts endothelial NO bioavailability, leading to endothelial dysfunction and enhanced expression of adhesion molecules. These changes facilitate circulating monocyte adhesion to the endothelium and subsequent transmigration into the intima. Within the subendothelial space, ROS/RNS drive LDL oxidation and ApoA-I nitration, promoting monocyte differentiation into macrophages and polarization toward a pro-inflammatory M1 phenotype. Sustained oxidative and inflammatory signalling enhances foam cell formation, vascular smooth muscle cell (VSMC) proliferation, and extracellular matrix remodelling, ultimately contributing to atherosclerotic plaque development and progression. Figure generated using BioRender.

In elevated cardiovascular risk conditions like type 2 diabetes and obesity, peripheral blood monocytes show increased ROS generation due to ER stress and metabolic dysregulation, contributing to accelerated atherosclerosis [173,174]. Similarly, smoking exposure intensity correlates with H_2_O_2_ production by monocytes, providing a mechanistic link between this risk factor and endothelial dysfunction [175]. At the molecular level, multiple sources contribute to ROS overproduction in atherosclerotic plaques. Nox enzymes, particularly Nox2 and Nox4, are major contributors, with their expression upregulated by pro-inflammatory cytokines and oxLDL [176,177]. Mitochondrial dysfunction also plays a critical role, as demonstrated by persistent monocytic bioenergetic impairment and mitochondrial DNA damage in patients with post-acute sequelae of COVID-19 with cardiovascular complications [147]. Environmental factors such as fine particulate matter (PM2.5) further exacerbate oxidative stress by inducing direct and macrophage-mediated vascular endothelial dysfunction [178] (Figure 4).

RNS, particularly ONOO•^−^, contribute to atherosclerosis by nitrating tyrosine residues in proteins, thereby altering their function. Nitration of apolipoprotein A-I (apoA-I) impairs its cholesterol efflux capacity, while nitration of prostacyclin synthase reduces its vasoprotective effects. Additionally, RNS can induce S-nitrosylation of key signalling proteins, modulating inflammatory pathways and contributing to plaque vulnerability [179,180]. The interplay between ROS and RNS creates a vicious cycle of oxidative damage that promotes necrotic core formation and plaque rupture, the precipitating event in most acute coronary syndromes [181,182]. The redox milieu within atherosclerotic plaques critically influences macrophage polarization. M1 macrophages, which dominate in unstable plaques, produce high levels of ROS and pro-inflammatory cytokines, further exacerbating inflammation and tissue damage [183,184]. In contrast, M2 macrophages, which are more prevalent in stable plaques, exhibit enhanced antioxidant defenses and promote tissue repair through the production of IL-10 and TGF-β [185,186,187]. The balance between these subsets is regulated by redox-sensitive transcription factors such as NF-κB and Nrf2, with oxidative stress favouring M1 polarization and impairing the transition to M2 phenotypes [188,189] (Figure 4).

Additionally, chronic oxidative stress in atherosclerosis does not only modify polarization but also leads to DNA damage and accelerated cellular senescence in monocytes and macrophages. Increased ROS production and DNA damage in monocytes serve as biomarkers of both aging and atherosclerosis, reflecting cumulative oxidative injury [171]. Senescent macrophages exhibit a senescence-associated secretory phenotype (SASP), characterized by increased secretion of pro-inflammatory cytokines and matrix metalloproteinases, which contribute to plaque destabilization [190].

**Figure 5:**
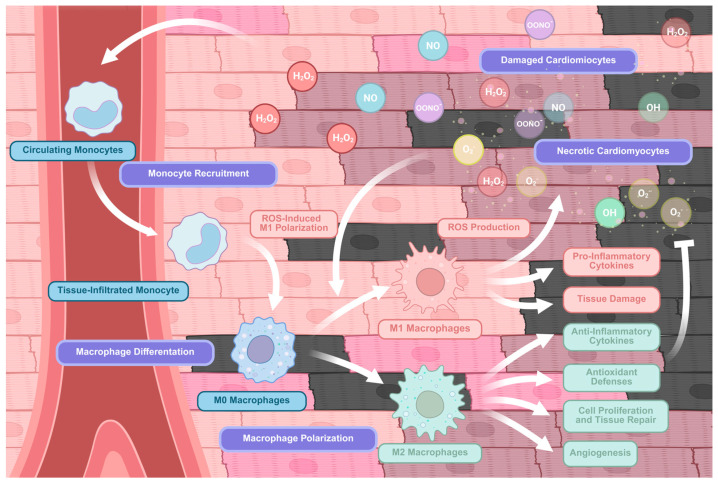
ROS/RNS modulating monocyte and macrophage behaviour in the ischaemic myocardium. Ischaemic injury to the myocardium triggers excessive generation of ROS/RNS, including O_2_•^−^, H_2_O_2_, •OH, NO•, and ONOO•^−^, released by damaged and necrotic cardiomyocytes as well as infiltrating immune cells. Elevated ROS levels promote monocyte recruitment from the circulation and their infiltration into the injured tissue, followed by differentiation into macrophages. Within the ischaemic myocardium, redox signalling critically regulates macrophage polarization: high and sustained oxidative stress favours pro-inflammatory M1 macrophage polarization, leading to increased ROS/RNS production, secretion of pro-inflammatory cytokines, and amplification of tissue damage. Conversely, controlled ROS/RNS levels support the transition toward anti-inflammatory and reparative M2 macrophages, which secrete anti-inflammatory cytokines, enhance antioxidant defences, promote angiogenesis, and facilitate cell proliferation and tissue repair. The dynamic balance between ROS/RNS-driven inflammatory and reparative pathways determines the extent of myocardial injury and the efficiency of post-ischaemic healing. Figure generated using BioRender.

Upon rupture of an atherosclerotic plaque and subsequent coronary occlusion, resulting in myocardial ischemia and necrosis, circulating monocytes are rapidly recruited to the injured tissue, attracted by DAMPS and ROS, where they play a pivotal role in modulating the inflammatory and reparative response [191]. Depending on local microenvironmental cues, monocytes can differentiate into pro-inflammatory M1 macrophages, which are activated by signals such as IFN-γ, TNF-α, and DAMPs, leading to NF-κB- and MAPK-mediated transcription of pro-inflammatory cytokines and the generation of ROS, thereby amplifying oxidative stress and tissue injury. Conversely, differentiation into pro-resolving M2 macrophages, induced by IL-4, IL-13, and other anti-inflammatory mediators, promotes the activation of Nrf2 and STAT6 pathways, enhancing antioxidant responses, extracellular matrix remodelling, and angiogenesis, ultimately facilitating tissue repair and the resolution of oxidative stress [8,54,192]. This dynamic balance between M1 and M2 polarization, tightly regulated by redox signalling, is critical in determining the extent of myocardial damage and the efficiency of post-infarction repair (Figure 5).

## 7. Therapeutic Perspectives

The recognition of ROS and RNS as central mediators in monocyte and macrophage dysfunction has spurred significant interest in developing therapeutic strategies that modulate redox balance to treat inflammatory diseases, particularly atherosclerosis and its complications. Among the most studied approaches are antioxidant interventions, which aim to neutralize excessive ROS or enhance endogenous antioxidant defences. Vitamin C (ascorbic acid) has shown promise in this context, as demonstrated by studies showing that its intake reduces the increased adhesiveness of isolated monocytes to endothelium in smokers, potentially mitigating early steps in atherogenesis [193]. Interestingly, other studies have demonstrated how vitamin C exhibits a dose-dependent effects on monocytes profile, shifting from pro-oxidative to antioxidant and modulating monocytic cell morphology and protein modification to protect against oxidative damage [194]. Moreover, other antioxidants like vitamin E and carotenoids have been shown to prevent lymphocyte DNA damage induced by PMA-stimulated monocytes, suggesting a systemic protective effect that extends beyond innate immune cells [195]. The impact of antioxidant supplementation extends to modulation of cytokine networks, which are critically regulated by redox balance. Studies have demonstrated that antioxidants can significantly alter cytokine production profiles from monocytes, potentially shifting the balance from pro-inflammatory to anti-inflammatory states [196]. This immunomodulatory effect is particularly relevant in chronic inflammatory conditions where persistent oxidative stress drives maladaptive cytokine production. Vitamin D represents another promising nutraceutical approach, as its signalling has been shown to increase NO• and antioxidant defences in bovine monocytes, with similar effects likely in human cells. The pleiotropic effects of vitamin D on immune function, combined with its redox-modulating properties, position it as an attractive candidate for adjunctive therapy in cardiovascular and autoimmune diseases [197].

Beyond single antioxidants, research has increasingly focused on bioactive compounds from natural sources that offer synergistic antioxidant effects. Plant-derived polyphenols, flavonoids, and other phytochemicals have demonstrated remarkable capacity to modulate oxidative stress in monocytes and macrophages [198]. For instance, combinations of natural compounds show synergistic antioxidant effects on H_2_O_2_-induced cytotoxicity in human monocytes, providing a more robust protection than individual agents [199]. These compounds often work through multiple mechanisms, including direct free radical scavenging, metal chelation, and upregulation of endogenous antioxidant enzymes such as superoxide dismutase, catalase, and glutathione peroxidase.

While nutritional and nutraceutical approaches show promise, pharmacological interventions targeting specific ROS sources are gaining traction as more precise therapeutic strategies. Inhibitors of NOX, particularly selective inhibitors for Nox1, Nox2, and Nox4 isoforms, have demonstrated efficacy in reducing oxidative stress and inflammation in preclinical models of ischaemic disease [200]. These agents offer the advantage of targeting pathological ROS production without eliminating beneficial ROS signalling. Similarly, mitochondrial-targeted antioxidants such as Mito-Esc accumulate specifically in mitochondria, neutralizing ROS at their source while preserving physiological redox signalling elsewhere in the cell [201].

Emerging therapeutic frontiers also include modulation of redox-sensitive transcription factors and pathways. Activators of the Nrf2 pathway, such as sulforaphane and bardoxolone methyl, enhance the expression of a broad array of antioxidant and cytoprotective genes, offering a comprehensive approach to bolster cellular defences [202,203]. Conversely, inhibitors of the NF-κB pathway, such as salsalate and specific IKK inhibitors, can dampen the pro-inflammatory effects of ROS without completely blocking essential immune functions [204]. Additionally, strategies targeting the NLRP3 inflammasome, which is activated by ROS, have shown efficacy in reducing inflammation in experimental models [205,206].

Personalized medicine approaches will likely be crucial for the success of redox-modulating therapies. Genetic polymorphisms in antioxidant enzymes, variations in ROS production capacity, and differences in disease endotypes all influence individual responses to these interventions [207,208]. Biomarkers such as monocyte mitochondrial DNA damage, plasma levels of oxidized lipids, and imaging of plaque inflammation may help identify patients most likely to benefit from specific antioxidant or redox-modulating treatments [209,210]. Furthermore, combining redox-modulating agents with established therapies, such as statins, which have both lipid-lowering and pleiotropic antioxidant effects, may yield synergistic benefits [211].

A specific field in which antioxidant therapy may be particularly relevant is the modulation of oxidative alterations in monocytes and macrophages induced by anti-cancer treatments. ROS occupy a fundamentally paradoxical position in oncology: while they contribute to tumour initiation and progression by driving genomic instability, metabolic reprogramming, and chronic inflammation, they are simultaneously exploited by many chemotherapeutic agents and radiotherapy protocols as the primary mechanism of antitumoral cytotoxicity [212,213,214]. This duality has been extensively characterized, with ROS acting as Janus-faced molecules whose net effect on tumour biology and immune responses depends critically on their concentration, source, and cellular context [215]. In the tumour microenvironment, ROS exert complex and often opposing effects on anticancer immunity: at moderate levels they can enhance immune surveillance and promote immunogenic cell death, while at high or sustained levels they suppress effector immune responses and drive immune evasion [216,217]. A particularly striking mechanism through which ROS subvert antitumour immunity is the upregulation of PD-L1 on macrophages: oxidative stress directly modulates the immunosuppressive phenotype of tumour-associated macrophages by inducing PD-L1 expression, thereby dampening T cell responses and facilitating tumour immune escape [218]. Collectively, these observations highlight that while high ROS generation is central to the antitumoral efficacy of many treatment modalities, the oxidative reprogramming of circulating monocytes and tissue macrophages induced by therapy may promote persistent pro-inflammatory polarization, mitochondrial dysfunction, and impaired efferocytosis, thereby contributing to systemic inflammation, tissue damage, and long-term cardiovascular or metabolic complications in cancer survivors. Carefully calibrated antioxidant or redox-modulating strategies could therefore help preserve monocyte–macrophage homeostasis without compromising antitumour efficacy, representing a promising adjunctive approach in cardio-oncology and immuno-oncology settings, though the precise therapeutic window for such interventions remains a critical and as yet incompletely resolved challenge.

However, current approaches trying to modulate oxidative stress through generic antioxidant supplementation have repeatedly fallen short of their theoretical promise, and this gap between preclinical rationale and clinical reality demands a more critical appraisal. A fundamental conceptual problem is that indiscriminate ROS scavenging fails to account for the essential physiological roles of reactive species in immune signalling, host defence, and cellular homeostasis, roles that are thoroughly documented throughout this review. Dietary antioxidants have been shown to fail in protecting against oxidative genetic damage under controlled in vitro conditions, and supplemental antioxidants do not ameliorate inflammatory disease development even in contexts of severely depleted endogenous antioxidant reserves, as demonstrated in experimental colitis models with profoundly low mucosal glutathione levels [219,220]. More broadly, the clinical translation of antioxidant strategies has been consistently disappointing: large randomized trials of vitamins C and E in cardiovascular disease have failed to demonstrate benefit and, in some cases, have revealed potential harms, a paradox that has been attributed to the disruption of redox-dependent signalling cascades that are required for normal vascular and immune function [221]. This ambivalence is captured in the framing of antioxidants as potentially both positive and negative actors, whose net effect depends critically on dose, timing, cellular context, and the specific ROS species being targeted [222,223,224]. In the context of inflammatory immune-related diseases specifically, the relationship between antioxidant intervention and clinical outcome is similarly complex, with evidence suggesting that the timing, source, and selectivity of antioxidant action are at least as important as the magnitude of ROS neutralization [225,226]. Taken together, these observations make a compelling case that the future of redox-targeted therapy does not lie in broad-spectrum antioxidant supplementation, but rather in precisely targeted strategies that selectively modulate pathological ROS sources, such as specific Nox isoforms or dysfunctional mitochondria, while preserving the physiological redox signalling that is indispensable for monocyte and macrophage function.

Looking forward, the field must address several challenges to translate these promising approaches into clinical practice. These include developing more specific and potent redox modulators, identifying optimal timing and duration of interventions, and establishing reliable biomarkers to monitor target engagement and therapeutic efficacy. Additionally, understanding the complex crosstalk between redox signalling and other immunometabolic pathways will be essential for designing integrated therapeutic strategies that address the multifactorial nature of diseases like atherosclerosis. By overcoming these challenges, redox-modulating therapies have the potential to become valuable additions to our armamentarium against chronic inflammatory diseases, offering new hope for patients with conditions driven by oxidative stress and immune dysregulation.

## 8. Conclusions and Future Directions

Oxidative stress fundamentally shapes monocyte and macrophage biology, modulating their survival, differentiation, and polarization through redox-sensitive signalling pathways. These reactive species act as essential regulators of the balance between inflammation and tissue repair, with critical implications in both health and disease.

Despite significant advances, key gaps remain in understanding how oxidative stress differentially affects monocyte subsets and drives macrophage plasticity. Future research must address these questions using innovative approaches like single-cell analyses and advanced redox imaging to fully elucidate the complexity of redox regulation in innate immunity.

Looking ahead, redox-immunomodulatory therapies represent a promising frontier. Moving beyond broad antioxidants, strategies targeting specific ROS sources, enhancing endogenous defences, or combining redox modulation with metabolic interventions offer new opportunities to restore immune balance. Success will require personalized approaches guided by redox biomarkers and interdisciplinary collaboration to translate basic science into effective treatments for chronic inflammatory, infectious, and degenerative diseases.

## Data Availability

No new data were created or analyzed in this study. Data sharing is not applicable to this article.

## Abbreviations

The following abbreviations are used in this manuscript:

AMPK	AMP-activated protein kinase
AP-1	Activator protein 1
ApoA-I	Apolipoprotein A-I
ATP	Adenosine triphosphate
Akt	Protein kinase B
CCR2	C-C chemokine receptor type 2
CX3CR1	C-X3-C motif chemokine receptor 1
DAMPs	Damage-associated molecular patterns
DNA	Deoxyribonucleic acid
ER	Endoplasmic reticulum
ERO1α	ER oxidoreductase 1-alpha
GTPases	Guanosine triphosphatases
GSTs	Glutathione S-transferases
H_2_O_2_	Hydrogen peroxide
HIF-1α	Hypoxia-inducible factor 1-alpha
HMGB1	High-mobility group box 1
HO-1	Heme-oxygenase-1
IFN-γ	Interferon-gamma
IKK	Inhibitor of nuclear factor kappa-B kinase
IL-1β	Interleukin-1 beta
IL-4	Interleukin-4
IL-6	Interleukin-6
IL-10	Interleukin-10
IL-13	Interleukin-13
iNOS	Inducible nitric oxide synthase
IRAK	Interleukin-1 receptor-associated kinase
Keap1	Kelch-like ECH-associated protein 1
LPS	Lipopolysaccharide
M1	Classically activated macrophage
M2	Alternatively activated macrophage
MAPK	Mitogen-activated protein kinases
MCP-1	Monocyte chemoattractant protein-1
mTOR	Mechanistic target of rapamycin
MPO	Myeloperoxidase
MtSOD	Mitochondrial superoxide dismutase
NADPH	Nicotinamide adenine dinucleotide phosphate
NF-κB	Nuclear factor kappa-light-chain-enhancer of activated B cells
NLRP3	NACHT, LRR, and PYD domains-containing protein 3
NO•	Nitric oxide
Nox	NADPH oxidase
NQO1	NADPH quinone oxidoreductase 1
Nrf2	Nuclear factor erythroid 2-related factor 2
O_2_•^−^	Superoxide anion
• OH	Hydroxyl radical
ONOO•^−^	Peroxynitrite
oxLDL	Oxidized low-density lipoprotein
PAMPs	Pathogen-associated molecular patterns
PDI	Protein disulfide isomerase
PI3K	Phosphatidylinositol 3-kinase
PKC	Protein kinase C
PM2.5	Particulate matter with a diameter of less than 2.5 μm
PMA	Phorbol 12-myristate 13-acetate
PRR	Pattern recognition receptor
PTMs	post-translational modifications
RNS	Reactive nitrogen species
ROS	Reactive oxygen species
SASP	Senescence-associated secretory phenotype
STAT6	Signal transducer and activator of transcription 6
TLR	Toll-like receptor
TNF-α	Tumor necrosis factor-alpha
TRX	Thioredoxin
TXNIP	Thioredoxin-interacting protein
UCP2	Uncoupling protein 2
UPR	Unfolded protein response
VSMC	Vascular smooth muscle cell
